# Low-grade mucoepidermoid carcinoma mimicking benign cystic lesions in the salivary gland: A diagnostic dilemma

**DOI:** 10.1177/20363613241242397

**Published:** 2024-03-23

**Authors:** Wangpan Shi, Timothy Law, Kevin Thomas Brumund, Jennifer Chang, Charmi Patel, Grace Lin, Jingjing Hu

**Affiliations:** 1 Department of Pathology, 8784 University of California, San Diego, CA, USA; 2 Department of Pathology, 436933 California Northstate University, College of Medicine, La Jolla, CA, USA; 3 Department of Otolaryngology, 8784 University of California, San Diego, CA, USA; 4 Department of Radiology, 8784 University of California, San Diego, CA, USA

**Keywords:** Salivary gland tumor, low grade mucoepidermoid carcinoma, cystic degeneration, rare tumors, acinic cell carcinoma

## Abstract

Mucoepidermoid carcinoma (MEC) is a common malignancy arising in the parotid gland. The diagnosis of MEC is typically based on its morphological features alone, characteristically containing mucocytes, intermediate cells and epidermoid cells. However, when cystic degeneration is diffuse, it is challenging to distinguish MEC from other benign cystic tumors. This is a case report of a 58-year-old Caucasian man who presented with a parotid mass. H&E sections of the mass reveal multiloculated cysts lined by bland-looking epithelium with only rare papillary architectures. The papillary proliferation contains mucocytes, and epidermoid cells highlighted by the p63 immunohistochemistry study. The diagnosis was confirmed by FISH result of positive MAML2 (11q21) rearrangement. Patient underwent parotidectomy and is disease-free 6 months post-surgery. MEC with cystic degeneration is a common diagnostic pitfall which can mimic many benign lesions in the salivary gland. We present a rare case with MEC with extensive cystic change, its molecular and pathologic findings and review the diagnostic features of MEC, its benign mimickers and useful tools for distinguishing these entities.

## Introduction

Mucoepidermoid carcinoma (MEC), the most common type of salivary gland malignancy, usually manifests as a painless swelling mass predominantly located over the parotid glands, for tumor arising from minor salivary glands, the palate, buccal and gingival mucosa, floor of mouth are all possibly sites.^1–4^ There is a female predominance with an average onset at 49 years and a 5-years overall survival rate of 67% to 90%, depending on the histological grade.^1,5^ In general, MECs of low to intermediate grade can be adequately treated with surgical excision and have favorable outcomes. However, three major factors contribute to poor outcomes: perineural invasion, extracapsular lymph node extension, and lymph node metastasis.^6^ AFIP and Brandwein report that low-grade MEC is often well-circumscribed with cystic components admixed with rich mucous secreting cells.^3,7,8^ Nuclear atypia, mitosis, or tumor invasion are typically absent.^7,8^ Based on WHO Classification of Tumors 5th edition, MEC presents histologically with cystic and solid patterns containing mucinous, intermediate, and epidermoid cells.^9^ The identification of intermediate and epidermoid cells can be particularly challenging when there is extensive cystic degeneration. Therefore, differentiating MEC from benign cystic lesions can pose a challenge.^10^ Herein we describe an interesting case of low-grade MEC with extensive cystic components mimicking benign cystic lesions.

## Case presentation

**Figure 1.**
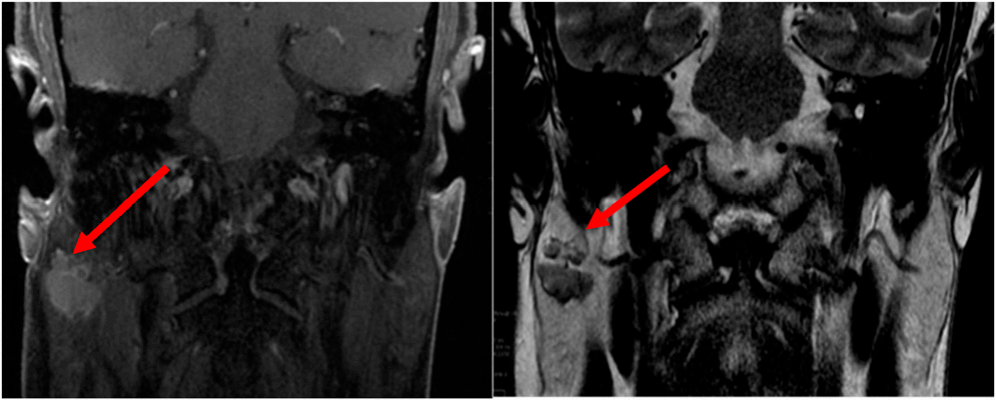
Incidental finding on head MRI with coronal postcontrast fat saturated image of the brain (left) and T2 weighted image of the head (right) demonstrating avidly enhancing bilobed T2 hypointense lesion within the right parotid gland.

This patient is a 58-year-old Caucasian male with a history of chronic intermittent tinnitus and hearing loss who was referred to otology. MRI IAC was ordered to evaluate possible retrocochlear pathology, and a hypointense right parotid lesion measuring 1.8 × 2.1 cm was incidentally discovered (Figure 1). Following a discussion of the risk, benefit, and alternatives of surgery such as fine needle biopsy (FNA), the patient favored surgery. Total right parotidectomy with facial nerve preservation was performed. Other than transient right-sided facial paresis, no post-operative complications were noted. No adjuvant treatment was applied after tumor board discussion.

**Figure 2.**
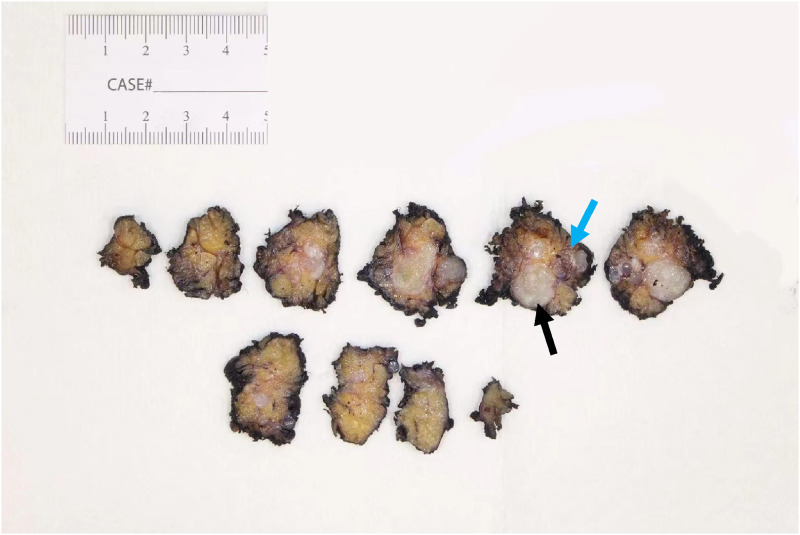
The specimen was serially sectioned to reveal a multiloculated white to tan solid-cystic mass. The cut surface shows a cystic component (blue arrow) and the white nodular area (black arrow). The mass abuts the inked outer surface.

**Figure 3.**
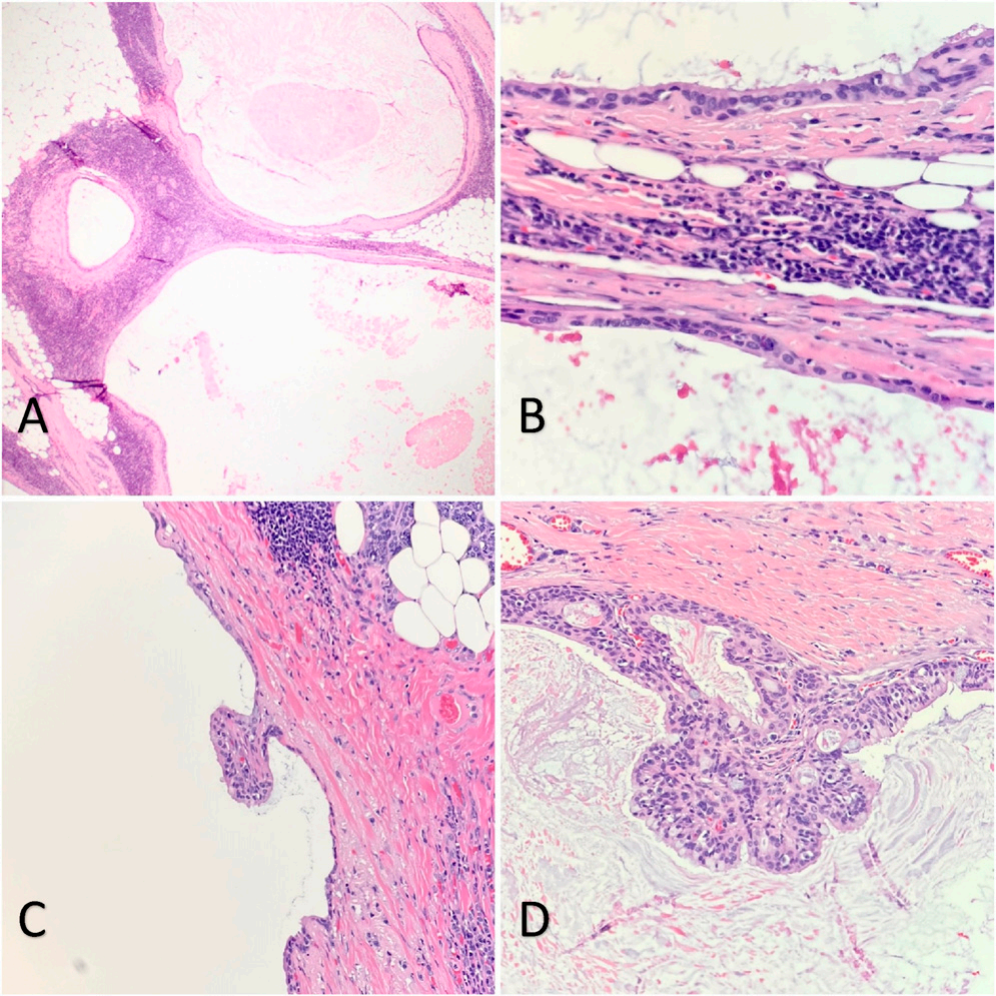
Histologic examination of the parotid mass shows multicystic lesions lined with bland epithelium containing pink amorphous material in the lumen (A, 40x; B, 200x) and ample lymphocytic infiltration in the stroma (B, 200x). There are multiple papillary infoldngs along the cyst walls with most devoid of mucous cells (C, 200x). However, several foci of mucinous-rich epithelium arranged in fused papillary structures are noted (D, 200x).

**Figure 4.**
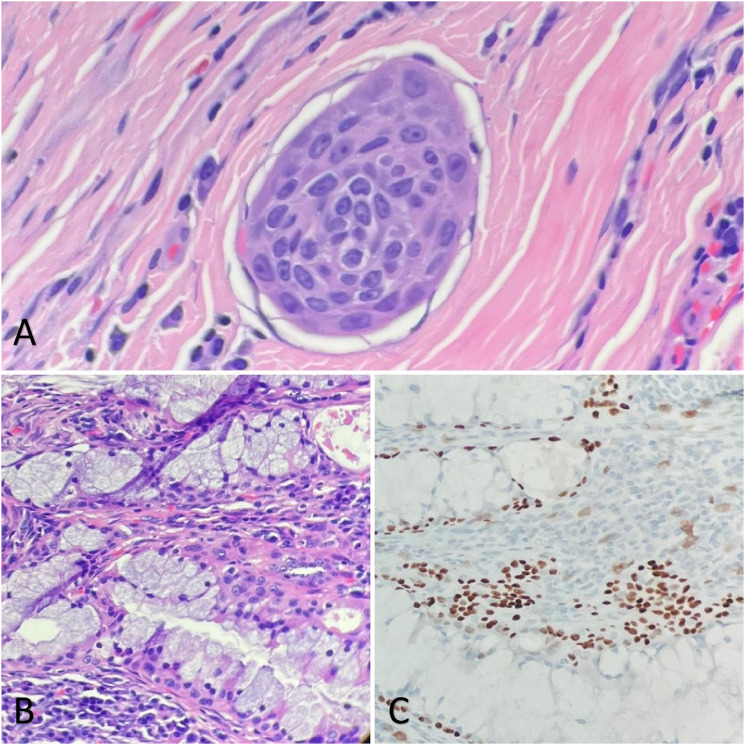
Tumor cells within an endothelial-lined structure is suspected, raising the possibility for lymphovascular invasion (A, 400x). The papilla with mucous cells also contains epidermoid cells on H&E (B, 200x) that stain positive for p63 (C, 200x) which supports the diagnosis of mucoepidermoid carcinoma.

Gross examination of the specimen revealed a multiloculated solid-cystic surface with yellow lobulated tissue in the periphery (Figure 2). On H&E section, multiple cysts lined by bland-looking cuboidal to columnar epithelium with lymphoid proliferation in the cystic wall are present (Figure 3(A) and (B)). No mitosis or necrosis are observed. Focal areas were lined by papillary infoldings (Figure 3, (C) and (D)) with scant to abundant mucous cells. Rare groups of intermediate cells with round to oval nuclei and open chromatin are seen (Figure 4 (B)). Lymphocytes and plasma cells are observed in the papillary stroma without well-formed germinal centers. P63 staining was performed to highlight the cyst wall lining and intermediate cell aggregates (Figure 4 (C)). An area of lymphovascular invasion was suspected, which suggested malignant nature (Figure 4 (A)). The resection margins were negative for tumor. FISH study demonstrated MAML2 (11q21) rearrangement, and the diagnosis of low-grade mucoepidermoid carcinoma was rendered. The patient has been regularly followed up for 2 years without further treatment, and no new complaints have been reported.

## Discussion

There are different grading systems established for mucoepidermoid carcinoma, according to the extent of aggressiveness.^3,7,8^ As a result of the subjectivity and heterogeneity of tumor differentiation in large resection specimens, no consensus has been reached. High-grade MEC is characterized by a solid growth pattern, fewer mucous cells, perineural or lymphovascular invasion, and conspicuous nuclear pleomorphism, along with mitotic figures. MECs of intermediate grade fall between the above criteria. Although the AFIP grading system promotes reproducibility, tumor aggressiveness may not directly correlate with grade as some low-grade tumors act more aggressively.^7,8^

MEC is typically diagnosed by its morphological features alone, and immunohistochemistry (IHC) is not required. The presence of P63 expression with the absence of S100 or SOX10 markers may help differentiate MEC from other salivary tumors.^11^ Microscopically, mucous cells appear columnar with abundant intracellular mucin. Epidermoid cells have rich eosinophilic cytoplasm with occasional formation of keratin pearls. Intermediary cells are characterized by smaller nuclei and cytoplasm compared to epidermoid cells.^12^ Oncocytic or clear cells may be present in some areas, and it is reported they can constitute the majority of tumor.^1^ As a result, MECs are classified as sclerosing, oncocytic, clear-cell, warthin-like, ciliated, spindle-cell, and mucoacinar, based on their highly diverse morphologies.^13–18^ Translocation at t (11;19) (q21;p13), forming the CRTC1::MAML2 fusion gene is the defining feature of most MECs.^19^ There are, however, very few instances of translocation at t (11;15) (q21;q26) with the CRTC3::MAML2 fusion.^20^

We presented a case with the initial complaint of hearing loss and subsequent finding of a right painless parotid mass. The presence of a well-circumscribed solid mass with multilocular cysts, positive p63 stain in epidermoid cells, and the FISH finding of MAML2 (11q21) rearrangement support the diagnosis of low-grade MEC. A particular highlight of our study is the low grade mucoepidermoid carcinoma is a great mimicker of benign cystic lesions/neoplasms.

There are many different etiologies that can present as a parotid mass with prominent cystic architecture, ranging from benign to malignant processes. There are three main categories:: cysts without malignancy, benign cystic tumors, and macrocystic tumors with malignant transformation.^21^ The list includes parotid duct cyst, mucinous cystadenoma, mucocele, infarcted pleomorphic adenoma, sialometaplasia, Warthin tumor, lymphoepithelial cyst, mucoepidermoid carcinoma and very rarely squamous cell carcinoma. Parotid duct cysts can be congenital or secondary to trauma or chronic inflammation.^22^ The majority of congenital parotid gland cysts involve the superficial lobe of the parotid gland, which is sometimes referred to as a branchial cleft cyst.^23,24^ In patients with a history of salivary duct obstruction, acquired parotid duct cysts are often suspected.^25^ Parotid duct cysts are usually lined with stratified squamous epithelium and subepithelial lymphoid tissue. Typically, complex papillary architecture is not observed along the cystic lining.^23^ There may be sparse to moderate lymphocytic infiltration of the cyst wall. In some cases, ductal obstruction leads to oncocytic metaplasia.^25^ Cystadenomas are well-circumscribed, benign tumors, half of which arise in the parotid gland, malignant transformation of benign cystadenomas can be seen in exceedingly rare occasions.^26, 27^ The tumor presents histologically as a multicystic lesion lined by proliferative epithelium with varying proportions of papillary architecture and oncocytic change.^28^ Fibrous stroma can sometimes contain foci of lymphocytic cell aggregates.^29^ In addition, several subtypes are described according to their unique diagnostic features. Mucinous cystadenoma is defined by a predominant mucinous columnar-lined cyst wall without papillary architecture.^30^ The cystic cavity is commonly filled with amorphous mucinous material. Mucocytes are always present, but mucinous cystadenomas typically do not harbor multiple cell populations alone the cystic lining.^30,31^ Cystadenomas can also be of the papillary oncocytic subtype, where the epithelium is lined with oncocytoid cells with prominent papillary architecture that can manifest as multicystic lesions.^32^ CK7, CK5/6, and mammaglobin may be positive, p63 highlights the myoepiethlial layer.^32^

Warthin tumors are benign tumors consisting of oncocytic epithelium arranged in cystic or papillary patterns overlying rich lymphoid stroma, seen most commonly in males ages 60 to 70.^33^ On gross examination, it can appear as a solid area within a multicystic lesion with admixed papillary projections.^34^ The cystic line is composed of the bilayered epithelium with a luminal layer of tall, columnar or oncocytic cells and basal triangular cells. A great mimicker is MEC, Warthin-like subtype. Unlike the aforementioned tumors, pleomorphic adenomas are rarely characterized by cystic changes at their initial stage although post-biopsy/procedure infarctions or necrosis can result in secondary degenerative cystic changes.^35^ Moreover, none of the three main components for diagnosis, ductal cells, myoepithelial cells, and chondromyxoid stroma, were identified in this case. Another differential diagnosis is acinic cell carcinoma, especially when presents with papillary cystic (macro-cystic) architectures. Microscopically, it shows papillary projection of tumors or a hobnailing morphology, where secretions can be observed in the cystic lumen.^36^ Acinar cells are large and polyhedral, with basophilic granular cytoplasm. The granules can be highlighted be diastase resistant positive periodic acid-Schiff (PAS) reaction. Positive DOG1 stain and negative for p63 are useful tools to make the discretion between other malignant salivary gland tumors.^37^

Lastly, lymphoepithelial cysts are another epithelial-lined cystic lesion affecting the salivary glands, with stratified squamous epithelium reported in most cases.^38^ Bilateral lymphoepithelial cysts are commonly seen in individuals who have contracted the human immunodeficiency virus (HIV).39 Fewer cases of mixed mucous and cuboidal-lined epithelium have been reported. It is characterized by lymphoid tissue with germinal centers along the stroma as well as the absence of cytological atypia.^38^ Squamous cell carcinoma, which is often metastatic from other sites (such as skin or mucosal origin), shows very prominent cytological atypia and lacks intermediate cells and mucocytes. It usually shows well-developed keratinization.

## Conclusion

Our study describes a diagnostic dilemma of low grade mucoepidermoid carcinoma with prominent cystic appearance mimicking benign cystic lesions. Careful examination of advanced papillary structures containing mucous cells is critical. However, due to the challenge in identifying epidermoid cells and intermediate cells across the extensive cystic background, p63 immunohistochemistry and molecular confirmation of MAML2 rearrangement are useful tools for establishing the correct diagnosis.
